# Working hour characteristics and schedules among nurses in three Nordic countries – a comparative study using payroll data

**DOI:** 10.1186/s12912-019-0332-4

**Published:** 2019-03-28

**Authors:** Anne Helene Garde, Anette Harris, Øystein Vedaa, Bjørn Bjorvatn, Johnni Hansen, Åse Marie Hansen, Henrik A. Kolstad, Aki Koskinen, Ståle Pallesen, Annina Ropponen, Mikko I. Härmä

**Affiliations:** 1 The National Research Center for the Working Environment, DK-2100 Copenhagen, Denmark; 2 Department of Public Health, Copenhagen University, DK-1014 Copenhagen, Denmark; 3 Department of Psychosocial Science, University of Bergen, N-5020 Bergen, Norway; 4 Department of Global Public Health and Primary Care, University of Bergen, N-5018 Bergen, Norway; 5 Danish Cancer Society Research Center, DK-2100 Copenhagen, Denmark; 6 Danish Ramazzini Centre, Department of Occupational Medicine, Aarhus University Hospital, DK-8200 Aarhus, Denmark; 7 Finnish Institute of Occupation Health, FI-00251 Helsinki, Finland

**Keywords:** Shift work, Quick returns, Night work

## Abstract

**Background:**

Organisation of working hour schedules in the Northern European countries are rather similar. EU countries are obliged to adopt national legislation regarding duration of weekly working hours and rest periods. Yet, working hour characteristics and schedules are likely to differ with respect to starting times and duration depending e.g. on culture and tradition. Yet, very little is known about potential differences between shifts and schedules across countries among nursing personel. This knowledge is relevant, since the potential differences in working hour characteristics may influence and possibly explain some of the differences observed in studies of health and safety.

The aim of the study was to compare characteristics of working hours and work schedules among nursing personel in three Nordic countries: Denmark, Finland and Norway.

**Methods:**

The study populations included nursing personnel holding a ≥ 50% position at public hospitals in Denmark (*n* = 63,678), Finland (*n* = 18,257) or Norway (*n* = 1538) in 2013. Objective payroll based registry data with information on daily starting and ending times were used to compare working hour characteristics e.g. starting time, duration of shift, and quick returns (< 11 h between two shifts), as well as work schedules e.g. permanent or 3-shift work between the three countries.

**Results:**

Night shifts generally started earlier and lasted longer in Finland (10–11 h starting at 20:00–22:59) than in Norway (10 h starting at 21:00–21:59) and in Denmark (8 h starting at 23:00–23:59). Very long shifts (≥12 h) were more common in Denmark (12%) compared to Finland (8%) and Norway (3%). More employees had many (> 13/year) quick returns in Norway (64%) and Finland (47%) compared to Denmark (16%). The frequency of 3-shift rotation workers was highest in Norway (41%) and lower in Denmark (22%) and Finland (22%). There were few differences across the countries in terms of early morning shifts and (very) long weekly working hours.

**Conclusion:**

Despite similar distribution of operational hours among nurses in the three countries, there were differences in working hour characteristics and the use of different types of work schedules. The observed differences may affect health and safety.

## Background

Compared to other European countries, working time regimes in the Northern European countries are rather similar with low weekly working hours and high working-time autonomy and workplace flexibility [1]. Finland and Denmark, but not Norway, are members of the European Union, and working hours are accordingly governed by EU’s Working Time Directive (2003/88/EC). EU countries are obliged to adopt national legislation to ensure that weekly working hours do not exceed 48 h on average including any overtime. Further, a minimum daily rest period of 11 consecutive hours every 24 h is enforced. Yet, there are also differences between countries in terms of how working hours are organized. In all Nordic countries, working hours are also regulated by union-based collective agreements [2]. These agreements also cover personnel who are not members of a trade union. Full time ranges from 37 h per week in Denmark, including 0.5 h lunch break in most public jobs, to 40 h per week including 0.5 h unpaid lunch for daytime workers in Norway [3]. In Denmark and Finland most nursing personnel work highly irregular shift systems, which are planned for 4–8 week periods in Denmark [4] and 3–4 week periods in Finland. In Norway, there are major differences between hospitals and some work schedules are planned for up to 52 weeks at a time. Schedules are typically announced with a notice of down to 2–4 weeks.

Hospitals require services and staffing around the clock, though there are differences in work load intensity between day and night work. To cover the need for staff at all times, the work is organized in shifts, e.g. day, evening and night shifts of different durations, which in turn are organized into schedules e.g. permanent or rotating (2- or 3-shift schedules) work. However, working hour characteristics and schedules are likely to differ with respect to starting times and duration depending e.g. on culture and tradition. Yet, very little is known about potential differences between shifts and schedules across countries. Such knowledge is relevant since the potential differences in working hour characteristics may influence health. Differences in night shift characteristics may also explain the heterogenity in results of studies on shift work with health [5]. Negative health effects of shift work have typically been associated with night shifts, but also with other shift characteristics such as the duration of the daily and weekly working hours, quick returns (time off work < 11 h), number of consecutive night shifts, and direction and speed of rotations [6–8]. Thus, it is well documented that working night shifts is associated with shorter and disturbed sleep, increased fatigue, occupational injuries, poor work performance, and higher work-life interference [9, 10]. Furthermore, many studies suggest that shift workers may have increased risk of cardiovascular disease, breast and prostate cancer, diabetes, and gastrointestinal disorders [11–15], but the role of the more specific shift characteristics associated with exposure to shift work is not well studied.

Working hour characteristics may be described in several ways depending on sources and purpose. An often used categorization of shift work is based on schedules e.g. whether the employee has only day, evening or night shifts or different combinations of these (e.g. day and evening shifts or day, evening and night shifts). Assessment of shift schedules is usually based on self-report. Survey methods have, however, the disadvantage that the classification is crude, leading to misclassification and specific characteristics of working hours cannot be disentangled. The establishement of new cohorts based on the starting and ending times of daily work shifts from objective payroll data of working hours has now facilitated the possibilities to use more precise exposure information on night and shift work [16, 17]. With this type of information it is possible to define precise working hour characteristics relevant for weekly work load, health and recovery.

## Methods

The aim of the present study was to describe and compare characteristics of working hours and work schedules among nurses in Denmark, Finland and Norway under consideration of potential health impact, and to develop a joint working hour terminology and codification for future studies of objective working hours and health. We aimed to answer the following research questions:

1. Are there differences between Denmark, Norway and Finland in the working hour characteristics in terms of timing and length of work shifts, quick returns, number of consecutive night shifts, and weekly working hours?
2. Are there differences between the countries in the prevalence of work schedules like permanent day, evening and night work vs. rotating 2- and 3-shift schedules?

### Populations and data sources

The study populations included nursing personnel holding at least a 50% position with at least one shift in a public hospital in 2013. Data sources comprised: The Danish Working Hour Database (DWHD) from Denmark, the Working Hours in the Public Sector (WHFPS) from Finland, and the Register study of Working hour, Health and Sickness absence (RWHS) from Norway.

*DWHD* contains payroll based daily working hour data from all employees in all the five Danish administrative regions [18]. The primary task of the regions is to run the public hospitals and handicap homes. In all, 10.5% of the original cohort had part-time work (< 50% position). For the current analysis we included nurses and assistant nurses in 2013 (*n* = 63,678) covering 9,726,640 shifts. This sample consisted of 88% women with a mean age of 42.9 (SD = 11.7) years. The database was approved for research by the Danish Data Protection Agency (2015-57-0074). The need for consent by participants is deemed unnecessary according to national regulations according to the Danish Dataprotection Lac, nr 502 af 23/05/2018.

*WHFPS* data in the present study included workers from five hospital districts and workers from one social health care department of one town (FPS hospital cohort). The data was retrieved from the historical records of the used (100% of the nursing personnel) shift scheduling programme Titania® (CGI Finland) [16]. A total of 8.5% of the original cohort had part-time work (< 50% position). For the current analysis we included nurses and assistant nurses in 2013 (*n* = 18,257) covering 2,939,711 shifts. The sample comprised 92% women with a mean age of 44.0 (SD = 11.1) years. The need for consent by participants is deemed unnecessary by the decision of the ethics committee of the Hospital District of Helsinki and Uusimaa that approved the FPS study (HUS 1210/2016).

*RWHS* comprises payroll based daily working hour data from employers’ records at Haukeland University Hospital in western Norway. Haukeland University Hospital is one of the four central hospital units in Norway with responsibility for some of the highly specialised health care functions in the country. Data were retrieved after obtaining consent to use the registry data from the individual participants. Holding a ≥ 50% position was a criterion for inclusion. A total of 1538 nurses and assistant nurses agreed to participate (response rate 41.5%). In total 233,545 shifts were included. Of the included participants, 88% were women and the mean age was 42.5 (SD = 12.0) years. The study was approved by the Norwegian Regional Ethics Committee (2013/526/REKnord) and the Norwegian Social Science Data Services (NSD, ID 34212). All participants gave written consent.

In all cohorts the data was based on administrative payroll data, i.e. data on working hours with detailed information on daily starting and ending times for shifts for all employees. Consecutive observations of working hours were combined into one registration defined by the starting time and ending time. Registrations 60 min or less apart were collapsed into one shift; i.e. gaps of 60 min or less were considered work time. Gaps of more than 60 min were considered time off and working hours prior and subsequent to the gap were recorded as two independent shifts. Thus there may be more than one shift per day. For the present study we included realized shifts and excluded on-call work in 2013.

### Working hour characteristics

Shifts were classified according to time of day: *Day shift* (starts after > 06:00 h and ends before < 21:00 (any duration); *Evening shift* (at least 3 h between ≥18:00 and < 02:00); *Night shift* (at least 3 h between 23:00 and 06:00 (both included)); *Early morning shift* (starts after > 03:00 and not later than 06:00 (any duration)) and duration: *Long shift* (≥ 9 h and < 12 h); and *Very long shift* (≥ 12 h and < 24 h). Some definitions of shifts were based on previous literature [16, 17] and legislation, others on discussions and consensus among the authors. The definitions of day, evening, night and early morning shifts are not mutually exclusive and each shift may be assigned several categories/exposures. In order to analyse the health risk the exposures were prioritized, so that each shift was assigned as either night (highest priority), evening or day (lowest priority). For example, in the Danish cohort the night shift category included was based on a combination of shifts defined as night shift only (9.8% of all shifts) and as both evening and night shift (27.8%) according to the aforementioned definitions.

Work patterns consisting of combinations of shifts were characterized as: *Quick returns* (< 11 h between two shifts); *Long weeks* (number of calendar weeks with > 40 h/week); *Very long weeks* (number of calendar weeks with > 48 h/week); and *periods of ≥ five consecutive night shifts*.

Characteristics of shifts and work patterns were summed by individual and grouped into categories. *Day shift* were divided into four categories: 0, 1–100, 101–200 and > 200 shifts/person/year; *Evening shift*, *Night shift*, *Early morning shift*, *Long shift*, *Very long shift*, *Long weeks*, *Very long weeks*, and *Quick returns* were also divided into four categories: 0, 1–12, 13–50, and > 50 per person/year, *periods of ≥ five consecutive night shifts* were divided in three categories: 0, 1–12 and > 12 per person/year.

Lastly, we looked at all shifts within the year for each person and classified the work schedules as permanent day (D), evening (E) or night (N) work, 2-shift work (D/E, D/N, or E/N) and 3-shift work (D/E/N). We used the following definitions based on the work by Härmä et al. [19], where a cut-off of 10 shifts out of 150 shifts per year (at least one shift in a month, corresponding to 6.7% on annual level) was used: *Permanent day*: ≥ 6.7% day shifts, < 6.7% evening shifts, and <  6.7% night shifts per year; p*ermanent evening*: < 6.7% D, ≥ 6.7% E, and <  6.7% N per year; *permanent night*: < 6.7% D, < 6.7% E, and ≥ 6.7% N per year; *day/evening:* ≥ 6.7% D, ≥ 6.7% E, and < 6.7% N per year; *day/night:* ≥ 6.7% D, < 6.7% E, and ≥ 6.7% N per year; *evening/night:* < 6.7% D, ≥ 6.7% E, and ≥ 6.7% N per year; and *day/evening/night:* ≥ 6.7% D, ≥ 6.7% E, and ≥ 6.7% N per year.

## Results

**Fig. 1:**
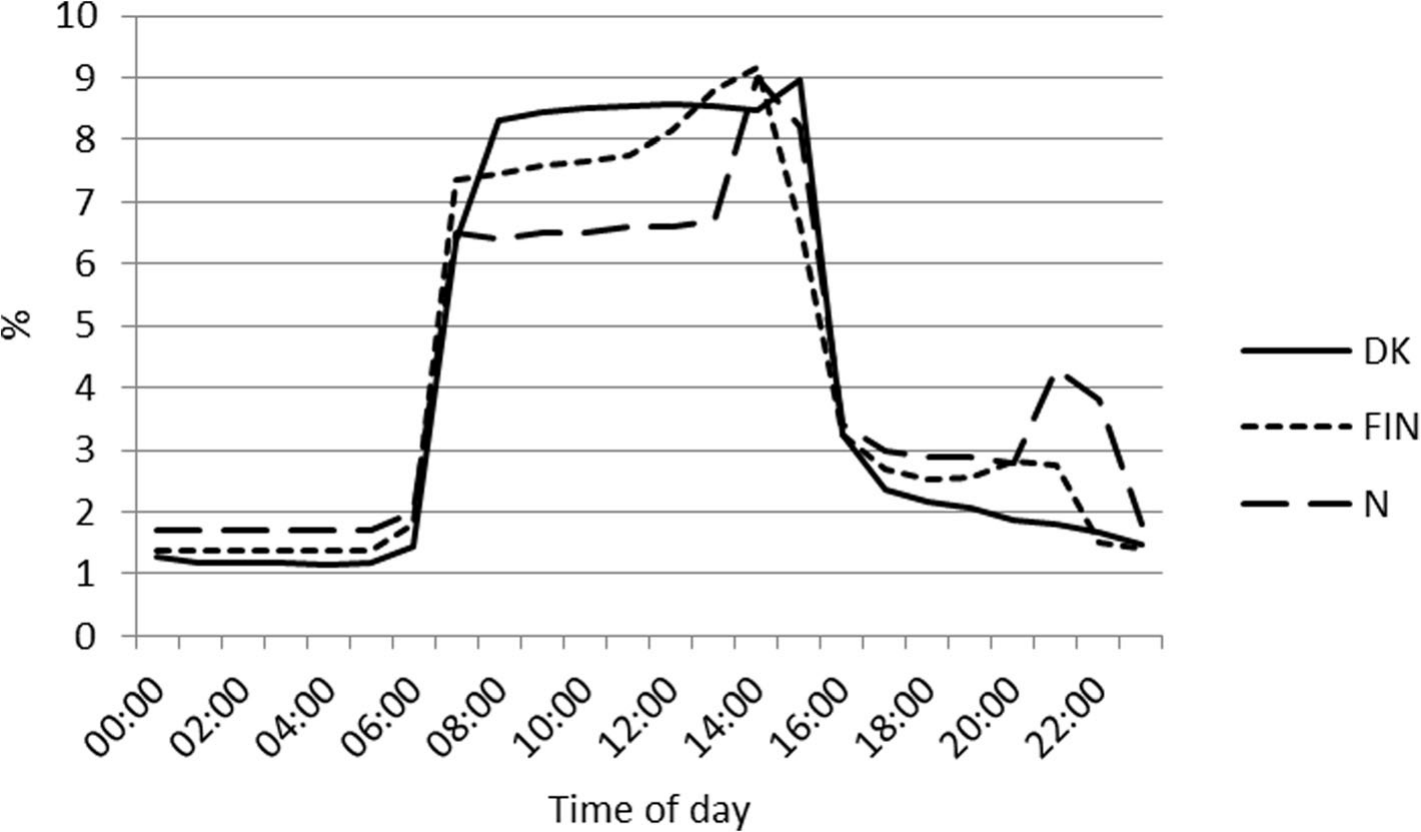
Percentage of operational working hours by time of day and country

Figure 1 shows the distribution of nursing personnel at work by time of day in the Danish, Finnish and Norwegian populations. In general, the distribution of nursing personnel across weekdays and weekends was rather similar in the Danish and Finnish populations, but slightly different in the Norwegian population. Thus, over a day most (7.9–9.4%) of operational hours were during the morning (8:00–13:00) in Denmark and Finland, whereas the corresponding number for Norway was lower (6.4–6.7%). The figure indicates that there were more operational working hours around 15:00, in Finland (9.4%) and Norway (9.0%), and around 21:00 in Norway with 4.3% compared to Denmark (8.2% at 15:00 and 1.8% at 21:00, respectively). The distribution of operational hours on different weekdays was similar across countries with 16.3–18.4% operational hours on Mondays-Thursdays, 15.1–15.9% on Fridays and 6.1–8.3% during weekends. Norway (8.3%) had the highest percentage of nurses at work during weekends, and Denmark (6.1%) the lowest.

Tables 1, 2 and 3 show the distribution of all shifts according to the start and duration of the shift in the three populations. In Denmark, the majority of shifts started between 7:00 and 7:59 (42.1%), 15:00–15:59 (12.6%) and 23:00–23:59 (7.8%). In Norway, the majority of shifts started between 7:00 and 7:59 (40.9%), 14:00–14:59 (21.5%) and 21:00–21:59 (13.7%). In Finland, 49.9% of shifts started between 7:00 and 7:59, 9.8% at 13:00–13:59, whereas starting times later in the day were more spread out with less than 6% starting within a given hour. In Denmark, the majority of night shifts started between 23:00 and 23:59 and lasted for 8 h. In Finland, most night shifts started between 20:00 and 21:59 and lasted 10 or 11 h. In Norway, most night shifts started between 21:00 and 21:59 and lasted 9 or 10 h.

**Table 1:**
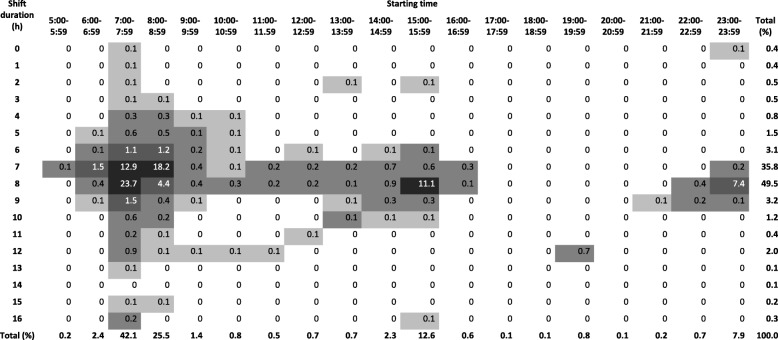
Distributions of shifts among nursing personel in Denmark

Distribution of shifts in DWHD (Denmark) according to start and length of shift among nurses and assistent nurses in public hospitals (*n* = 63,678). Darker color indicates higher percentage

**Table 2:**
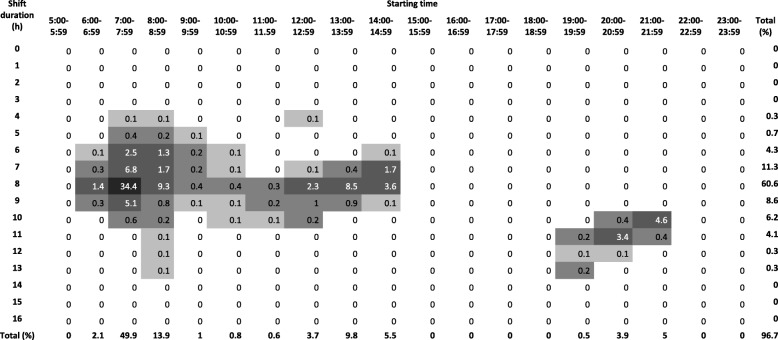
Distributions of shifts among nursing personel in Finland

Distribution of shifts in WHFPS (Finland) according to start and length of shift among nurses and assistent nurses in public hospitals (*n* = 18,257). Darker color indicates higher percentage

**Table 3:**
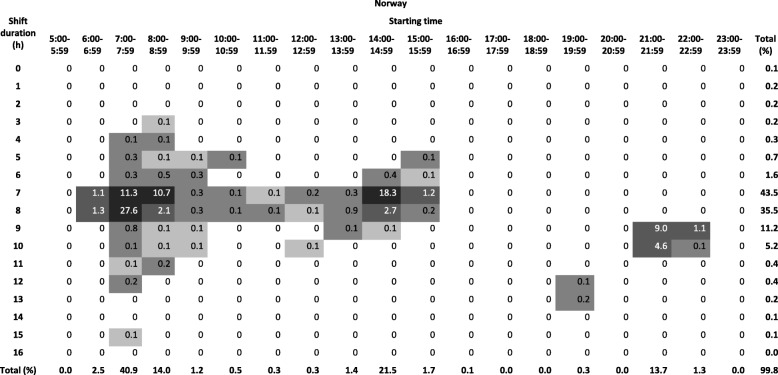
Distributions of shifts among nursing personel in Norway

Distribution of shifts in RWHS (Norway) according to start and length of shift among nurses and assistent nurses in public hospitals (*n* = 1538). Darker color indicates higher percentage

Table 4 illustrates the distribution of working hour characteristics summarized by individuals in the three populations. In Norway, more nurses (37%) had > 50 evening shifts per year compared to Denmark (21%) and Finland (12%). The number of nurses with > 50 night shifts per year varied from 10% in Finland and Denmark to 14% in Norway. Percentage of nurses with very long shifts were most frequent in Denmark (12%). In all, 64% of Norwegian nurses had more than 13 quick returns per year. In Denmark and Finland the corresponding numbers were 16 and 47%, respectively. In all three countries there were less than 6% early morning shifts. In Denmark and Finland less than 3% had five consecutive night shifts or more on a regular basis (> 12 times per year), whereas none of the Norwegian nurses had five consecutive night shifts or more. Number of nurses with (very) long weekly working hours was similar between countries.

**Table 4:** Distribution (%) of working hour characteristics summarized by individuals in the three countries

		DWHD (Denmark)	WHFPS (Finland)	RWHS (Norway)
Dayshift	0	2	1	2
	1–100	45	41	58
	101–200	41	45	36
	> 200	13	13	4
Eveningshift	0	32	42	16
	1–12	17	22	13
	13–50	30	24	34
	> 50	21	12	37
Nightshift	0	52	54	33
	1–12	20	13	20
	13–50	18	23	33
	> 50	10	10	14
Early morning shift	0	94	99	98
	1–12	6	1	2
	13–50	0	0	0
	> 50	0	0	0
Long shift (≥ 9 h < 12 h)	0	22	34	20
	1–12	42	33	28
	13–50	29	25	37
	> 50	7	8	15
Very long shift (≥ 12 h < 24 h)	0	55	56	69
	1–12	33	36	28
	13–50	11	7	2
	> 50	1	1	1
Quick returns (< 11 h between shifts)	0	37	24	17
	1–12	47	29	19
	13–50	15	42	58
	> 50	1	5	6
Long weeks (> 40 h/week)	0	38	61	23
	1–12	56	33	70
	13–50	6	6	7
	> 50	0	0	0
Very long weeks (> 48 h/week)	0	79	80	59
	1–12	21	20	41
	13–50	1	0	0
	> 50	0	0	0
Periods of ≥ 5 consecutive night shifts	0	93	85	94
	1–12	5	12	6
	> 12	2	3	0

*DWHD* The Danish Working Hour Database (Denmark), *WHFPS* The Working Hours in the Public Sector (Finland), *RWHS* the Register study of Working hour, Health and Sickness absence (Norway)

Table 5 shows that permanent day workers were less frequent in Norway (15%) and most frequent in Finland (46%). The frequency of permanent night workers ranged from 2.3% in Denmark to 5.3% in Norway. Workers with 2-shift schedules in terms of D/E was less frequent in Finland (18%) compared to the other countries, whereas the combination of D/N was more frequent (12%). In all three populations, less than 1% of the nurses were 2-shift workers with E/N according to our definitions. The frequency of 3-shift workers (D/E/N) was highest in Norway (41%), and lower in Denmark (22%) and Finland (22%).

**Table 5:** Distribution of workers with permanent day, evening or night work and and shift work

	DWHD (Denmark)	WHFPS (Finland)	RWHS (Norway)
Permanent day (%)	37.2	45.6	15.4
Permanent evening (%)	2.3	0.2	0.4
Permanent night (%)	2.3	2.4	5.3
Day/evening (%)	28.2	17.9	32.8
Day/night (%)	8.1	11.9	4.6
Evening/night (%)	0.8	0.3	0.4
Day/evening/night (%)	21.1	21.8	41.1
	100.0%	100.1%	100.0%

Definitions: *Permanent day*: ≥ 6.7% day shifts (D), < 6.7% evening shifts (E), and < 6.7% night shifts (N) per year; p*ermanent evening*: < 6.7% D, ≥ 6.7% E, and < 6.7% N per year; p*ermanent night*: < 6.7% D, < 6.7% E, and ≥ 6.7% N per year; *day/evening:* ≥ 6.7% D, ≥ 6.7% E, and < 6.7% N per year; *day/night:* ≥ 6.7% D, < 6.7% E, and ≥ 6.7% N per year; *evening/night:* < 6.7% D, ≥ 6.7% E, and ≥ 6.7% N per year; and *day/evening/night:* ≥ 6.7% D, ≥ 6.7% E, and ≥ 6.7% N per year

*DWHD* The Danish Working Hour Database (Denmark), *WHFPS* The Working Hours in the Public Sector (Finland), *RWHS* the Register study of Working hour, Health and Sickness absence (Norway)

## Discussion

The main findings of the present study were that there were substantial differences in working hour characteristics among nursing personnel in Denmark, Finland and Norway. There were only minor differences in the distribution of operational hours over the day, but there were more operational hours during the evening and night at the Norwegian hospital. Night and evening shifts and permanent night work schedules were most common in Norway compared to Denmark and Finland. However, none of the Norwegian nurses worked five consecutive night shifts, which was in contrast Danish and Finnish nurses. Night shifts were longer and more employees had quick returns (< 11 h between two shifts) in Norway and Finland compared to Denmark. In contrast, the use of very long work shifts (≥ 12 h) was more common in Denmark. There were few differences between the three countries in relation to early morning shifts, and (very) long weekly working hours.

The starting times and duration of typical shifts showed some similarities but also notably differences, particularly in relation to night shifts. In all three populations, the majority (> 40%) of day shifts among nurses started at 7:00, which is also the time where night shifts ended. In Denmark, there is a strong tradition for arranging the day into three 8-h shifts, whereas there is more variation in Finland and Norway. Night shifts in Finland and Norway started earlier (20:00–21:00) and lasted correspondingly longer (9–11 h) than night shifts in Denmark, where night shifts typically started at 23:00. Thus, the standard night shift was typically of longer duration in Finland and Norway, compared to Denmark. The increased risk for accidents is of primary concern since accident risk has been shown to increase with shift duration, especially above 8 h [20–22], possibly related to increased sleepiness/fatigue. It should be noted that factors such as workload and napping opportunity may moderate the relationship between night work and health outcome. The effects on the circadian rhythms and melatonin excretion, which are some of the suggested mechanisms for the association between night work and cancer [15, 23], may be the same in all three countries, since both long and short night shifts cover the period from midnight to 5:00.

More nurses with very long shifts were observed in Denmark compared to Finland, but the number of nurses with long work weeks was similar, which indicates that the long shifts were planned and not associated with overtime work. Long weekly working hours (> 55 h/week) have been associated with increased risk of diabetes, stroke and to some extent cardiovascular disease [24, 25] in some but not all studies, including a recent large Danish study [26–28]. Yet, long shifts are not necessarily associated with health impairments if the total worked hours are not increased. One such example is compressed work weeks where the hours worked per day are increased, whilst the days worked are decreased in order to work the standard number of weekly hours in less than 5 days. In a review on health outcomes of compressed workweeks, Bambra et al. found that there were no detrimental effects on self-reported health, whereas work-life balance was generally improved based on five prospective studies with a control group [29]. An intervention study by Lowden et al. [30] also found that a change from 8-h to 12-h shifts was associated with increased satisfaction with workhours, sleep, and time for social activities. It is argued that these results are possibly due to the shorter sequences of the workdays, the longer sequences of consecutive days off, the fewer types of shifts (easier planning), and the elimination of quick returns [30]. It should also be noted that freedom to choose particular hours of work may moderate the effect of shift on health [4, 31].

In the Norwegian sample, there were slightly more operational hours during the night. However, despite more nurses having night shifts and permanent night work in Norway compared to Denmark and Finland, none of the Norwegian nurses had periods of five or more consecutive night shifts, whereas this was the case for 2–3% of nurses in Denmark and Finland. The number of consecutive night shifts has been associated with risk of breast cancer [32] and it has been recommended to reduce number of consecutive night shifts in order to reduce risk of breast cancer [23]. This indicates that the potential risk of breast cancer may be reduced in Denmark and Finland without changing the number of night shifts.

In Norway four times more and Finland three times more employees had a high number of quick retuns (less than 11 h between two shifts) compared to Denmark. This is surprising since based on the European directive, time-off periods between the shifts should basically be 11 h or more. Short recovery time between the shifts due to quick returns is associated with reduced sleep durations, prolonged sleep onset latency, and increased sleepiness and perceived stress [33, 34], and more work-pesonal life conflicts [35] as well as more sick leave [36]. The fact that fewer nurses had quick returns in Denmark despite similar work tasks and operational hours indicates that it may be possible to arrange the working hours with fewer quick returns and thereby reduce potential health risk also in Norway and Finland.

In all three countries, there were only few nurses with very long weekly working hours (> 48 h per week) and only 2% in Danmark and Finland and none in Norway had five consecutive night shifts or more on a regular basis. Thus, the work schedules generally comply with the EU’s Working Time Directive (2003/88/EC) and the recommendations regarding arrangement of working hours in relation to risk of breast cancer [23].

The major strength of the present study was the use of objective payroll data allowing us for the first time to investigate working hour characteristics related to the single shift, work patterns covering several shifts, and schedules, e.g. permanent, 2-shift or 3-shift work across countries. In the future, the detailed information provides the opportunity to study the effects of different working hour charateristics on health and safety and thereby provide recommendations on how to best organize work around the clock. Last, but not least, the present study provides a taxonomy over several important shift schedule characteristics that may facilitate communication, use of definitions, and cross-study comparsions and as such advance the methodological basis of shift work research [37].

The definitions of shifts were not mutually exclusive. Thus a 24-h shift was classified as both a day, an evening shift and a night shift, because the worker was considered to be exposed to and experience the risks and inconveniences of all three types of shifts. This classification was used since different exposures may be relevant for different outcomes, e.g. evening shifts are more important for socially related outcomes, whereas health outcomes are more related to night shifts [10, 14, 15]. Since the primary focus of the present paper was on health outcomes, shifts with multiple exposures were classified (in prioritized order) as either night, evening or dayshift, respectively.

In addition to reflecting general differences between organisation of working hours between the three countries, the observed differences may also to some degree reflect differences between the cohorts. The Danish and Finnish cohorts are somewhat similar as they include all nurses from both large and small hospitals, though only the Danish cohort is nationwide. The Norwegian cohort was based on data from nurses in one smaller central hospital with selected responsibility for some of the highly specialised health care functions in the country, requiring emergency readiness during the night and weekends. The Norwegian sample is thus not similarly representative for the whole country as compared to the samples in Denmark and Finland. This may also explain the observed higher amount of evening and night shifts in the Norwegian sample.

## Conclusion

In conclusion, the developed joint codification for payroll data proved usefull for identification of differences in working hour characteristics among nursing personel in Denmark, Finland and Norway. Despite similar distribution of operational hours among nurses in the three countries, there were differences in working hour characteristics and the use of different types of work schedules.

## Abbreviations

DWHD: The Danish Working Hour Database (Denmark)
RWHS: The Register study of Working hour, Health and Sickness absence (Norway)
WHFPS: The Working Hours in the Public Sector (Finland)
